# Quantitative photoacoustic imaging study of tumours in vivo: Baseline variations in quantitative measurements

**DOI:** 10.1016/j.pacs.2018.12.002

**Published:** 2018-12-07

**Authors:** Márcia Martinho Costa, Anant Shah, Ian Rivens, Carol Box, Tuathan O’Shea, Efthymia Papaevangelou, Jeffrey Bamber, Gail ter Haar

**Affiliations:** a Joint Department of Physics and Cancer Research UK Cancer Imaging Centre, Division of Radiotherapy and Imaging, The Institute of Cancer Research, London and The Royal Marsden NHS Foundation Trust, Sutton, London, SM2 5PT, United Kingdom; b School of Immunology and Microbial Sciences, Guy’s Hospital, King’s College London, London, SE1 9RT, United Kingdom

**Keywords:** Photoacoustic imaging, Hypoxia, Head and neck subcutaneous tumours, Pimonidazole, Blood sO_2_, Hemoglobin

## Abstract

Photoacoustic imaging (PAI) provides information on haemoglobin levels and blood oxygenation (sO_2_). To facilitate assessment of the variability in sO_2_ and haemoglobin in tumours, for example in response to therapies, the baseline variability of these parameters was evaluated in subcutaneous head and neck tumours in mice, using a PAI system (MSOTinVision-256TF). Tumours of anaesthetized animals (midazolam-fentanyl-medetomidine) were imaged for 75 min, in varying positions, and repeatedly over 6 days. An increasing linear trend for average tumoural haemoglobin and blood sO_2_ was observed, when imaging over 75 min. There were no significant differences in these temporal trends, when repositioning tumours. A negative correlation was found between the percent decrease in blood sO_2_ over 6 days and tumour growth rate. This paper shows the potential of PAI to provide baseline data for assessing the significance of intra- and inter-tumoural variations that may eventually have value for predicting and/or monitoring cancer treatment response.

## 1 Introduction

The immature, tortuous and hyperpermeable vascular network often found in tumours may not provide sufficient nutrients and oxygen for rapid and uncontrolled cell proliferation, which can result in regions of tumour cell hypoxia, i.e. low oxygen concentration in the vicinity of tumour cells [1]. Temporal fluctuations in the hypoxic state also occur [2], on a time-scale of minutes or hours when they result from an efflux of red blood cells, or days when they are due to vascular network remodelling and angiogenesis [3].

Hypoxia is associated with increased resistance to chemo- and radiotherapy (as extensively reviewed by Rockwell et al. [4]), two commonly used clinical cancer treatments. Assessing the spatial and temporal variation in hypoxia levels could be predictive of a cancer therapy response and may therefore have a crucial role in treatment planning and adaptation [5,6]. Conventionally, oxygen needle electrodes have been used to assess hypoxia in patients, by directly measuring tissue partial oxygen pressure (pO_2_) [7]. However, needle electrodes are invasive and provide only a local value, which is not representative of the whole tumour. Techniques which use conventional non-invasive imaging have been developed to attempt to infer, not only the oxygenation levels, but also the spatial distribution of hypoxia. Several Positron Emission Tomography (PET) radiotracers are available [[8], [9], [10]], providing a noninvasive alternative, albeit with a relatively poor spatial resolution and high cost.

Unfortunately, the use of radioactive isotopes is also ill-suited to longitudinal studies due to radiation dose accumulation. Blood oxygenation level dependent (BOLD)-magnetic resonance imaging (MRI) [11] enables the measurement of relative blood oxygen saturation (sO_2_) increase during carbogen challenge compared to air-breathing. It has been shown that these measurements are in good spatial agreement with tissue pO_2_ measurements [[12], [13], [14]] and histological markers of hypoxia [15,16]. BOLD-MRI, however, has poor specificity for hypoxia, and does not provide absolute sO_2_.

Recently, the use of combined ultrasound (US) and optical imaging techniques, such as photoacoustic imaging (PAI), has been proposed as a method of non-invasively mapping blood sO_2_ [[17], [18], [19], [20]]. PAI can identify the unique spectral signatures of deoxy- (Hb) and oxyhaemoglobin (HbO_2_) [17]. The quantification of the local levels of each chromophore further allows mapping of blood sO_2_ distribution in the tissue, defined as the ratio of HbO_2_ to the total amount of haemoglobin (HbT, the sum of HbO_2_ and Hb) [21].

PAI has been used for hypoxia mapping pre-clinically, in subcutaneous [15,18,[21], [22], [23], [24], [25], [26]] and brain tumours [27], showing good agreement with pimonidazole staining [18,22], an exogenous hypoxia marker [15], and gold nanorods [28], and imaging modalities, such as bioluminescence [20], high resolution and dynamic contrast enhanced US [21,23] and BOLD-MRI [24,25].

PAI provides a powerful tool for quantifying tumour blood sO_2_ and has the potential of being used in clinic to predict and monitor treatment response involving changes in tumour vasculature. A potential limitation is that variability resulting from the measurement technique, or the intrinsic biological variability, may obscure changes resulting from a treatment. Primary sources of variability could be technical (system hardware, positioning of the tumour with respect to imaging transducer, data processing) and/or biological (effect of anaesthesia, tumour growth). It is known that anaesthetic agents significantly alter haemodynamic parameters. For example in mice, isoflurane, urethane, pentobarbital sodium, or ketamine-xylazine anaesthesia are known to cause substantial effects on haemodynamic parameters [29]. Such variability needs to be considered when interpreting changes in tumour vasculature in response to therapies. Joseph et al. [30] performed a technical validation study of a PAI tomography system (MSOT-inVision 256-TF, iThera Medical), demonstrating both reproducibility and repeatability of the measurements in phantoms and in vivo. They showed that the position of the test object with respect to the transducer had the greatest impact on signal repeatability. Joseph et al. also monitored the influence of inhaled isoflurane on sO_2_ measurements in kidney and spleen. This present paper aims to characterise the biological variability of photoacoustic imaging measurements of Hb, HbO_2_, HbT and sO_2_ in a subcutaneous head and neck tumour model, commonly hypoxic, using the same commercial PAI tomography system. Short-term intrinsic biological variability, for a period of 75 min, was tested to investigate the effect of anaesthesia on tumoural sO_2_ and HbT. The tumour positions (with respect to the imaging transducer) were also altered to understand the impact on signal variability. Long-term variability in the parameters of growing tumours was assessed over a period of 6 days. Our results demonstrated the importance of assessing such tumoural baseline measurements prior to performing studies involving prediction and monitoring of treatment response, involving a change in sO_2_ and HbT.

## 2 Methods

### 2.1 Tumour model and growth rates

All research was conducted under the Guidelines of Animal Welfare provided by the UK Home Office with approval from the local animal welfare and ethics board and under Helsinki Declaration of 1975.

The human head and neck squamous cell tumour model, CAL^R^, used for this study is a noncommercially available cancer cell line developed at the Institute of Cancer Research [31].

Half a million cells were injected subcutaneously into the right flank of 6 week old female FOXnu^n1^ mice (∼25 g). Animals were monitored for the development of a mass. Once palpable, tumour dimensions were measured every 2 or 3 days, in 3 orthogonal directions (length (l), width (w) and height (h)), using Vernier calipers. Tumour volume (V) was calculated assuming an ellipsoidal shape, as commonly used for subcutaneous tumours, according to Eq. (1).

(1) $$V=\frac{1}{6}\times l\times w\times h$$

The volume error (V_error_), for each individual tumour, was estimated from the derivative of an ellipsoidal volume, assuming a measurement error (E_m_) of 0.5 mm in each direction, using Eq. (2).

(2) $$V_{error}=\sqrt{\left({\left(\frac{dV}{dl}\times E_{m}\right)}^{2}+{\left(\frac{dV}{dw}\times E_{m}\right)}^{2}+{\left(\frac{dV}{dh}\times E_{m}\right)}^{2}\right)}$$

Tumour dimensions are therefore presented as mean volume plus standard deviation of the estimate, based only on the accuracy of the three orthogonal dimension measurements, ignoring any deviation in shape from an ellipsoid.

Tumour growth curves were fitted to the Exponential-Linear Model for calculating tumour growth rates [32]. Two rates were calculated: α_0_ is the growth rate during the exponential phase, and α_1_ is the growth rate in the linear phase. For details of these calculations, see Supplementary Methods.

### 2.2 Photoacoustic imaging data acquisition and reconstruction

Mice were imaged using a commercially available, real time, multispectral optoacoustic tomographic (MSOT) device, inVision 256-TF small animal scanner (iThera Medical GmbH, Munich, Germany) [29]. The system has a tunable optical parametric oscillator pumped by a Q-switched Nd:YAG near-infrared laser. A 256 element, toroidally focused, ultrasound imaging transducer (4 cm radius) with a centre frequency of 5 MHz (60% bandwidth) was used to acquire the acoustic signal, resulting from the optical tissue excitation. These elements cover an angle of 270° around the tumour to create a cross-sectional image.

Tumours were first imaged (defined as Day1) when they reached a volume of approximately 200 mm^3^. Anaesthesia was induced using an intraperitoneal injection of midazolam:fentanyl:medetomidine (5.0:0.05:0.5 mg kg^−1^). This anaesthetic combination is known to provide long anaesthesia times (∼60 min) [33]. It is suitable for studies involving protocols which could potentially cause pain (e.g. therapies involving tissue heating) or where mice need to be re-anaesthetised multiple times over a short period (e.g. multimodality imaging, imaging-therapy studies), as will be performed by us as a part future work. Mice were placed horizontally in a holder encased in a thin (17 μm) polyvinyl chloride (PVC) film (see Supplementary Fig. S1), with their tumour facing vertically downwards, i.e. directly in the direction of the laser beam, and acoustically coupled to the membrane using ultrasound imaging gel. In order to provide acoustic coupling to the MSOT, the animal holder was completely submerged in a tank filled with 34 °C distilled water. During immersion, medical air (21% oxygen) and/or 100%-oxygen were supplied continuously to the mouse via tubing, in order to avoid asphyxiation. Imaging started approximately 5 min after placing the holder in the water tank, in order to allow the animal’s body temperature to equilibrate with the water’s temperature.

For acquiring the photoacoustic data, a volume of interest (VOI), containing the whole tumour was selected to avoid a large dataset. Transverse (medial-lateral direction) imaging slices were acquired at 1 mm intervals in the cranial-caudal direction. The optical imaging wavelengths used were: 700, 715, 730, 750, 760, 800, 850, and 900 nm. This wavelength range maximises the differences in Hb and HbO_2_ signals. Ten signal averages per wavelength were obtained for each imaging slice.

Photoacoustic images were reconstructed using a model-based inversion algorithm [34] which was implemented in the viewMSOT software (v3.8) provided by iThera Medical.

**Fig. 1:**
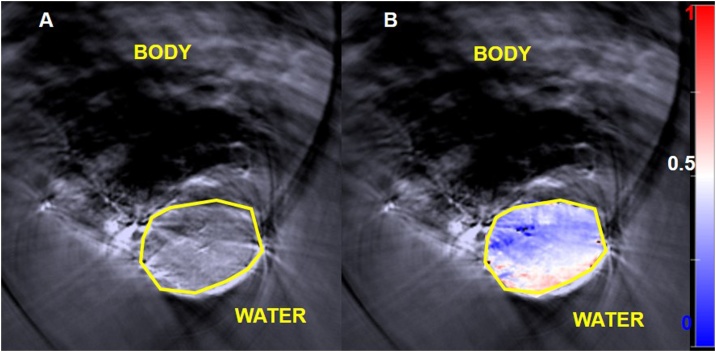
A: Greyscale photoacoustic image obtained from optical excitation at a single wavelength (800 nm). The yellow polygon is the ROI used to delineate the tumour. B: Shows an ‘oxymap’ overlaid on the tumour. The ‘oxymap’ colour scale on the right represents the blood sO_2_ calculated by the MSOT software on a pixel-by-pixel basis. Blue represents sO_2_ <0.5 A.U (white), while red is sO_2_ >0.5 A.U. Black pixels within the ROI show where haemoglobin signals are below the noise threshold.

Spectral unmixing was used to calculate the contribution of each chromophore, HbO_2_ and Hb, in blood, in order to calculate the sO_2_. This is done on a pixel-by-pixel basis, using a linear regression algorithm [35]. Pixels which indicated regions where the total amount of haemoglobin is so low, or sources of noise so high, that adequate confidence in spectral recognition could not be achieved, or where the reconstruction method produced pixels with negative Hb and HbO_2_ values, were set to a zero value. The software subsequently allows calculation of the mean HbO_2_ and Hb signals (which includes the zero value pixels) in a user defined region of interest (ROI) which could be drawn onto the greyscale anatomical image (Fig. 1A). These mean Hb and HbO_2_ values are added together to calculate the mean total haemoglobin per ROI. For any ROI the mean oxygen saturation was calculated using Eq. 3.

(3) $${mean\;sO}_{2}=\frac{mean\;{HbO}_{2}}{mean\;{HbO}_{2}+mean\;Hb}$$

All the parameters are unitless quantities, described in arbitrary units (A.U.), because the software does not use light fluence correction and the system response in calculations, as would be necessary to quantify the concentration (in mmol/L or mg/L) of each chromophore. The viewMSOT software also allows mapping of the spatial distribution of sO_2_ calculated on a pixel-by-pixel basis, obtaining an ‘oxymap’ (Fig. 1B). This was not used for calculating the mean sO_2_ values in ROIs because it was observed that the software did not account for the zero value pixels when calculating the mean values of sO_2_ in a region of interest. These pixels are discarded from the analysis. For each 3D imaging scan, 3 slices were always chosen for the analysis of Hb, HbO_2_, HbT and sO_2_.

### 2.3 Imaging studies

Three separate imaging studies were performed.

The first was a short-term MSOT study designed to investigate any potential effects of anaesthesia on HbT and blood sO_2._ A 3D whole tumour dataset was acquired every 5 min for 75 min, after allowing 5 min for the tumour to reach thermal equilibrium with the imaging system. Only air-breathing imaging was acquired, in order to maximise the rate (and number) of datasets that could be acquired. Measurements were acquired in 4 animals when their tumours reached a target volume of 200 mm^3^.

The PAI optical fluence is depth-dependent, and hence the position of tissue of interest with respect to the imaging transducer can affect the PAI measurements, as this can alter the amount of near-infrared light attenuated by overlying tissue, as well as the field intensity incident on that position. In order to study how re-positioning the animal affects measurements of blood haemoglobin and blood sO_2_, a second short term study was performed, 24 h after the first study. The same 4 animals were anaesthetised once and imaged in three consecutive sessions after being dismounted from the imaging system between each session. In addition, the tumour was placed at a slightly different position relative to the transducer each time. Whole tumour 3D datasets were acquired first under air breathing conditions which took approximately 2 min, and then under 100% oxygen breathing, after allowing 2 min for the oxygen to reach the tumour, for the same VOI, under hyperoxic conditions [25]. Oxygen breathing can be used as a contrast agent for PAI, enhancing tumour and spatial differences when vascularity is imaged using sO_2_ [36]. Approximately 4 min were needed to remove the animal from the holder, reposition it and replace it.

For both short-term studies, a linear regression model was applied to calculate an estimate of the average change in signal over time, as well as the goodness of fit between the imaging data and the model. This was obtained using the curve fitting toolbox (version 3.5.3) available in MATLAB R2016a software. To investigate the suitability of the linear model for these studies, three parameters were calculated: goodness of fit (R^2^) that indicates how much of the variability of the parameter (Hb, HbO_2_, HbT and sO_2_) is time-dependent; p-value of the model which shows if the slope is significantly different from 0 (if p-value<0.05); and the Sum of Squared Errors (SSE) of the prediction model that indicates the suitability of the linear model for explaining the variability behaviour of the data (the closer SSE is to 0, the better the linear fitting). The temporal variations of both the 75 min and the re-positioning study were also compared by calculating if there were statistical differences in signal change between studies, using an analysis of covariance (software: GraphPad Prism 7).

The third, a long-term study, was designed to image tumours sequentially during a period of growth. When tumours (n = 10) reached a volume of approximately 200 mm^3^, animals were imaged on 3 consecutive days (‘Day 1, 2 or 3′). This volume was chosen as being suitable for future studies of cancer treatment e.g. using radiotherapy. Tumours were also imaged on day 6 in order to evaluate changes in haemoglobin and blood sO_2_ during tumour growth. For this study, animals were imaged under air- and oxygen-breathing conditions.

For short- and long-term studies, three slices in each tumour, separated by 1 mm, were chosen for analysis, a central slice and the adjacent slice on either side. The mean haemoglobin and blood sO_2_ values were obtained by averaging over the three whole-tumour ROIs to provide the tumour ROI-averaged Hb, HbO_2_, HbT and sO_2_. For each animal, the average percentage of black pixels in the three central tumour slices was also calculated in order to evaluate the signal lost in each ROI. For the repositioning and long term studies, ΔsO_2_, i.e., the difference in blood sO2 between air- and oxygen-breathing was also calculated, as it has been shown as a useful parameter in other PAI published literature [25,36].

### 2.4 Statistical analysis

The slice-, intra-tumour and inter-tumour coefficients of variation (CoVs), i.e. the ratio of standard deviation to mean, were calculated for Hb, HbO_2_, HbT and sO_2_ in all PAI studies. Intra- and inter-tumour CoVs of ΔsO_2_ were also calculated for the repositioning and 6-day studies. The first indicates how much variation to expect between the 3 ROIs chosen for the analysis of the PAI parameters; the second the variation of the measurements for each animal and the third the variation between animals.

A paired student *t*-test was used to assess the level/degree of significance of differences in the mean Hb, HbO_2_, HbT and sO_2_ of CAL^R^ tumours during the long-term variability studies, on imaging days 1, 2, 3 and 6, using R software.

## 3 Results

### 3.1 Short term PAI variability study

#### 3.1.1 Variation in time: 75 min

**Fig. 2:**
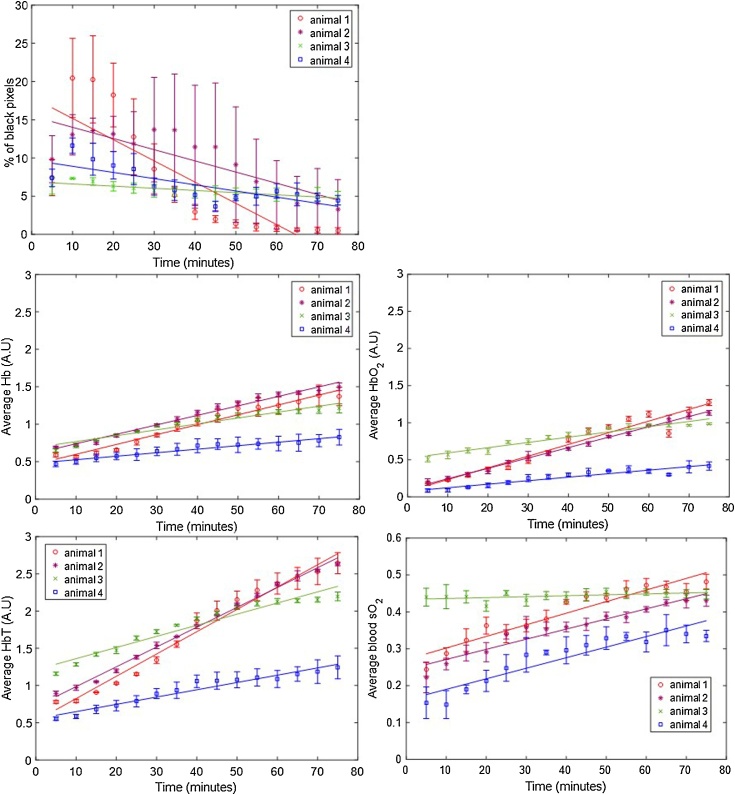
ROI-averaged percentage of black pixels, and Hb, HbO_2_, HbT and sO_2_ signals, for CAL^R^ tumours (n = 4) imaged over 75 min, during air-breathing. Error bars represent the standard deviation over 3 adjacent central tumour slices (ROIs), 1 mm apart. Solid lines represent a linear regression fitted to the data.

For the first short-term study, aimed at investigating the effect of anaesthesia over 75 min in the photoacoustic imaging measurements, four animals were imaged. Fig. 2 shows the short term variation in the ROI-averaged percentage of black pixels and average Hb, HbO_2_, HbT and sO_2_, over the three tumour slices per animal. A trend of a decrease in the percentage of black pixels (i.e. those with undetectable levels of haemoglobin), is observed for animals 1, 2 and 4 after the initial 10 min of imaging, after an initial increase between 5 and 10 min. Animal 3 has a more constant percentage of black pixels compared to the remaining 3 animals, over the whole imaging time. An increase in Hb and HbO_2_ (and therefore HbT), and sO_2_ over the whole time period was observed for all tumours, except for the sO_2_ values measured for animal 3.

**Fig. 3:**
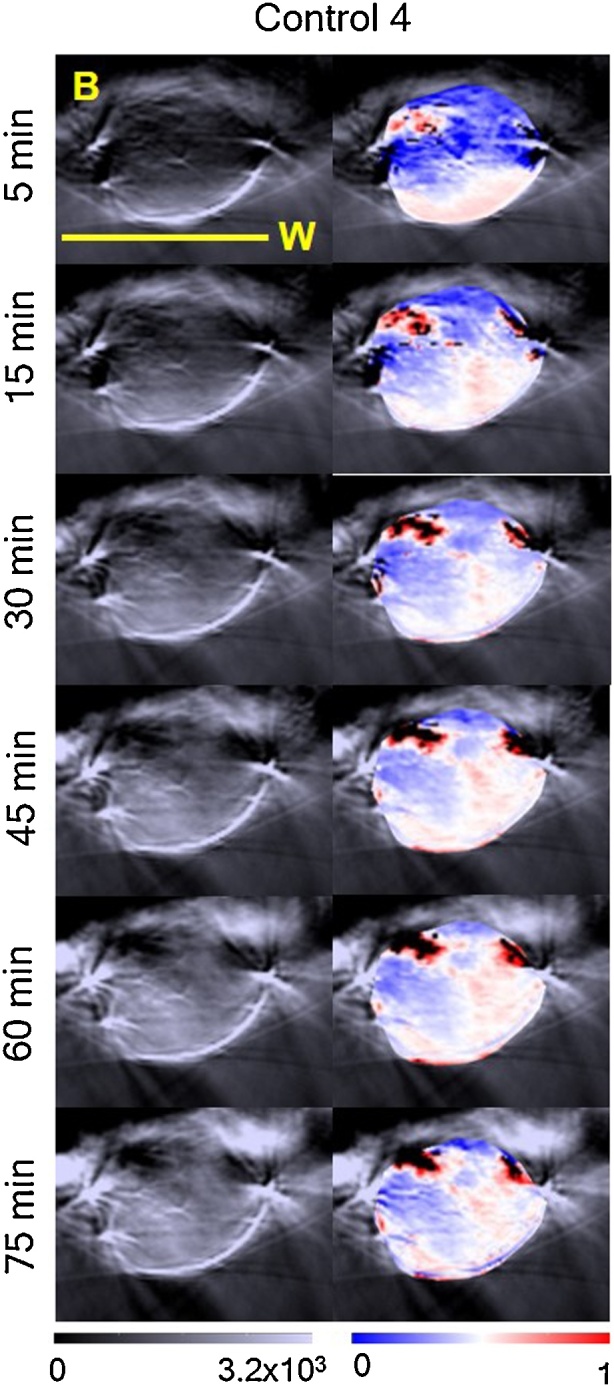
sO_2_ distributions or ‘oxymaps’ of one tumour, at 5, 15, 30, 45, 60 and 75 min after placing the animal in the tank. The ‘oxymaps’ are shown as overlays on greyscale photoacoustic images, of the central slice of each tumour. B: body; W: water. Yellow bar represents a 10 mm size scale.

Fig. 3 shows an example of the oxygenation maps obtained for this study. A radial sO_2_ gradient, but over time, more white and pink regions (blood sO_2_≥0.5) extend towards the middle of the tumour, and the margins attached to the body shift from dark blue to light blue, consistent with the indication of increased blood sO_2_.

The different change rates are shown in Table 1. This summarises the ROI-averaged rate of change of Hb, HbO_2_, HbT, sO_2_ and percentage of black pixels (%BP), as well as the goodness of fit (R^2^) and the sum of squares of error (SSE) of the linear regression used to calculate these rates of signal change. Animals 1 and 2 had a faster increase in Hb (and HbO_2_ than animals 3 and 4. This was reflected in the rate of HbT over 75 min. While for animals 1 and 2, the increase in HbT was >0.027 min^−1^, for animals 3 and 4, this rate was <0.015 min^−1^. Animals 1 and 2 also had a faster decrease in %BP (-0.278 min^−1^ and -0.146 min^−1^, respectively) compared to animals 3 and 4 (-0.0284 min^−1^ and -0.081 min^−1^, respectively).

**Table 1:** Rate of change of ROI-averaged Hb, HbO_2_, HbT, sO_2_ and percentage of black pixels (%BP) signal during the short-term (75 min) longitudinal study. The goodness of fit, R^2^, indicates how much of the signal variability is time dependent and the p-value whether the signal change (slope of the linear model) is significantly different from zero. The SSE reflects how similar the predicted values and measured data are, i.e. the closest SSE is to zero, the better the linear fit.

Animal		Signal change, min^−1^ (95% CI)	R^2^	p-value	SSE
1	Hb HbO_2_ HbT	0.013 (0.012, 0.015) 0.016 (0.013, 0.018) 0.030 (0.027, 0.033)	0.968 0.932 0.976	<0.001 <0.001 <0.001	0.037 0.117 0.142
	sO_2_	0.0031 (0.0025, 0.0038)	0.895	<0.001	0.007
	%BP	−0.278 (-0.392, -0.163)	0.679	<0.001	255
2	Hb HbO_2_ HbT	0.013 (0.013, 0.014) 0.014 (0.013, 0.015) 0.027 (0.026, 0.028)	0.981 0.995 0.995	<0.001 <0.001 <0.001	0.020 0.006 0.027
	sO_2_	0.0027 (0.0023, 0.0032)	0.923	<0.001	0.004
	%BP	−0.146 (-0.203, -0.0896)	0.704	<0.001	63
3	Hb HbO_2_ HbT	0.0079 (0.0066, 0.0091) 0.0071 (0.0060, 0.0082) 0.015 (0.013, 0.017)	0.930 0.934 0.938	<0.001 <0.001 <0.001	0.030 0.023 0.096
	sO_2_	0.00025 (3.148e-05, 0.00047)	0.267	0.049	0.001
	%BP	-0.0284 (-0.037, -0.020)	0.799	<0.001	1
4	Hb HbO_2_ HbT	0.0047 (0.0040, 0.0054) 0.0047 (0.0038, 0.0057) 0.0098 (0.0084, 0.011)	0.940 0.895 0.940	<0.001 <0.001 <0.001	0.009 0.017 0.039
	sO_2_	0.0029 (0.0022, 0.0035)	0.857	<0.001	0.009
	%BP	−0.081 (-0.118, -0.043)	0.622	<0.001	28

Table 1 also shows the increase in blood sO_2_. Animals 1, 2 and 4, had similar increases in blood sO_2_ (range: 0.0027-0.0031), but for animal 3, the blood sO_2_ rate of change was negligible, 0.00025 (3.148e-05, 0.00047), with R^2^ = 0.267. This is a consequence of a similar increase in Hb (0.0079 min^−1^) and HbO_2_ (0.0071 min^−1^).

The average slice-CoV, intra- and inter-tumour CoVs in ROI-averaged Hb, HbO_2_, HbT and sO_2_, over the 75 min acquisition for each animal, are summarised in Table S1. Overall, the intra-tumour CoV was higher for the haemoglobin components (Hb: 22.5±6.0%; HbO_2_: 40.2±13.9%; HbT: 22.9±9.2%) than for sO_2_ (16.0±8.4%). The slice-CoV, indicative of the variation of photoacoustic imaging parameters, in the 3 ROIs chosen for each tumour, was lowest for all the parameters (<13.9 ± 7.4%) compared to the intra-tumour CoV.

The inter-tumour CoV calculated for this study was 20.9 ± 3.1% for Hb, 46.4 ± 12.4% for HbO_2_, 28.6 ± 2.3% for HbT and 21.8 ± 10.9% for sO_2_. The largest variation was consistently measured for HbO_2_. These results show that, for the short-term longitudinal study, inter- and intra-tumour CoV were similar, showing a high variability is expected for each animal when imaged over 75 min.

#### 3.1.2 Re-positioning study

**Fig. 4:**
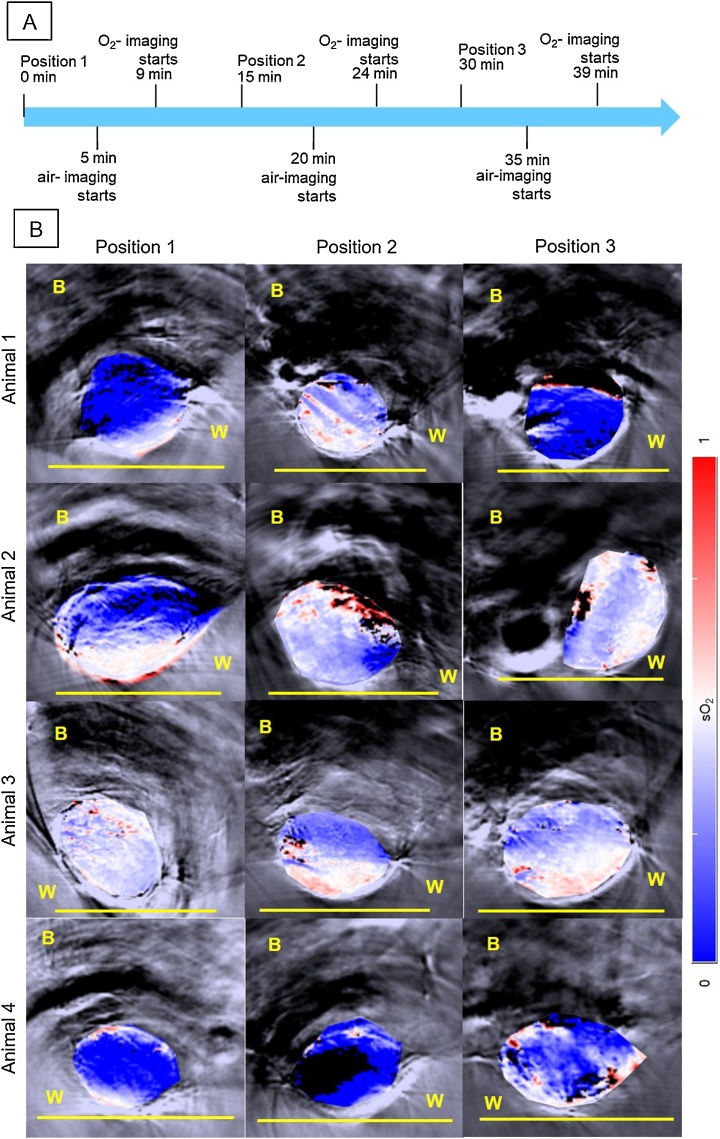
A: Diagram for the re-positioning of the animal. Each air-breathing imaging session started at approximately 5, 20 and 35 min after placing the animal in the water tank of the MSOT system. Air-breathing imaging takes approximately 2 min. The air is then changed for 100%-O_2_, for 2 min and the same tumour region is re-imaged for approximately 2 min again, during O_2_-breathing. B: sO_2_ distributions or ‘oxymaps’ of 4 CAL^R^ tumours, positioned differently by remounting the animals 3 times in the MSOT tank with the minimum time interval between each imaging session. The ‘oxymaps’ are shown as overlays on greyscale photoacoustic images, of the central slice of each tumour. B: body; W: water. Yellow bar represents a 10 mm size scale.

**Fig. 5:**
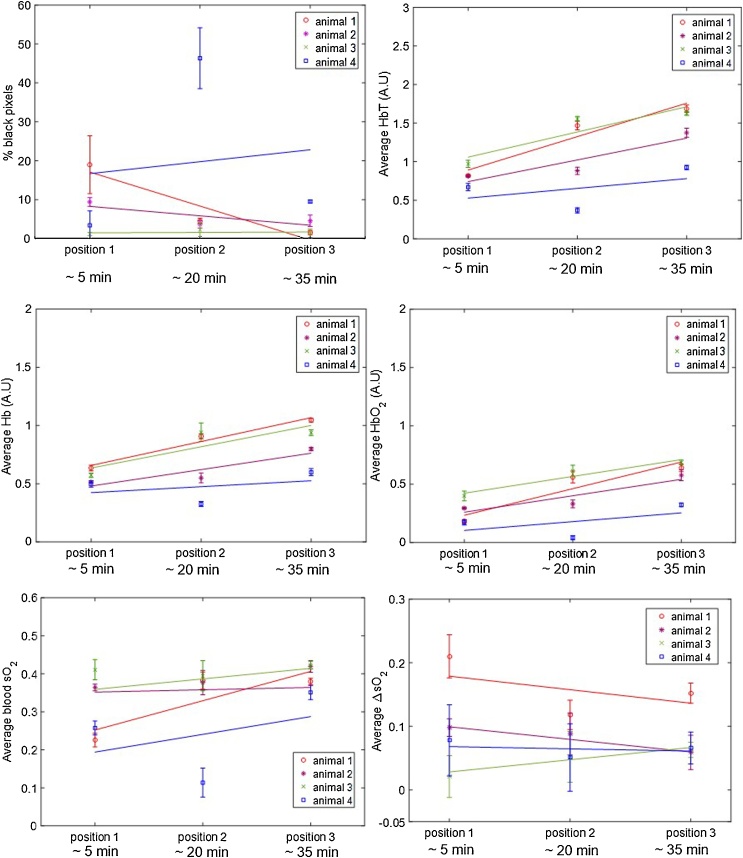
ROI-averaged percentage of black pixel, and Hb, HbO_2_, HbT, blood sO_2_ and ΔsO_2_ signals, for CAL^R^ tumours (n = 4) imaged after being removed and re-positioned in the tank three times, during air-breathing. Error bars represent the standard deviation over 3 adjacent central tumour slices, 1 mm apart. Solid lines represent a linear regression fitted to the data.

The results for the study on the effect of repositioning 4 animals three times in close succession in the photoacoustic system are shown in Fig. 4, Fig. 5. The trends for air- and oxygen-breathing were similar, so the results for air-breathing are shown here and the results for oxygen-breathing are in the Supplementary material (Fig. S2 and Tables S2–3). The ‘oxymap’ of the central tumour slice for each mounting position for each CAL^R^ tumour is shown overlain on greyscale anatomical images in Fig. 4. The images suggest a blood sO_2_ gradient radially into the tumour, ie, the margin below the skin tends to have higher blood sO2, compared to centre and margin attached to the mouse flank. The shapes and sizes of tumours also differ between positions. On the bottom row, it is possible to see that the blood sO_2_ of animal 4 is low, and in position 2, the ‘oxymap’ of the central slice of the tumour shows an atypically large region of black pixels (41%).

Following the changes observed in the ‘oxymaps’ in Fig. 4, the temporal changes in the ROI-averaged Hb, HbO_2_, HbT, sO_2_ and percentage of black pixels, were analysed, in order to investigate whether re-positioning had an additive effect to the time the animal is under anaesthesia in the temporal variability of the parameters analysed.

Fig. 5 shows the ROI-averaged percentage of black pixels for each animal, in each of the three imaging positions, for the 3 central slices. While there was some variations between animals and different positions, for the majority of the tumours the mean percentage of black pixels (undetectable haemoglobin levels) was <19%. Fig. 5 also shows that re-positioning the animals resulted in significantly different measurements of haemoglobin content and blood sO_2_ levels. A trend for an increase in ROI-averaged Hb, HbO_2_ and HbT was observed for most animals over time, independent of the starting and ending angle of the tumour relative to the transducer. ROI-averaged blood sO_2_ tended to increase for animals 3 and 4 but remain more constant for animals 1 and 2. The ΔsO2 values tended to decrease over time, apart from animal 3.

The quantification of the changes in signal, both for the individual haemoglobin components, blood sO_2_ and percentage of black pixels, is shown in Table 2. As observed in the 75 min imaging study, a trend for an increase in Hb (>0.0034 min^−1^), HbO_2_ (>0.0050 min^−1^) and HbT (>0.0084 min^−1^) was observed over time. The p-values in Table 2 show that the slopes, i.e. changes in signal over time, for Hb, HbO_2_, HbT and SO_2_, were not significantly different from zero.

**Table 2:** Rate of change of ROI-averaged Hb, HbO_2_, HbT, blood sO_2_, ΔsO_2_ and percentage of black pixels (%BP) signal during the re-positioning study (air-breathing). The goodness of fit, R^2^, indicates how much of the signal variability is time dependent and the p-value if the signal change (slope of the linear model) is significantly different from zero. The SSE reflects how similar the predicted values and measured data are, i.e. the closest SSE is to zero, the better the linear fit.

Animal		Signal change, min-1 (95% CI)	R2	p-value	SSE
1	HbT %BP Hb	0.030 (-0.077, 0.14) -0.582 (-3.38, 2.21) 0.014 (-0.019, 0.047)	0923 0.875 0.966	0.179 0.231 0.120	0.031 22 0.003
	HbO_2_	0.015 (-0.058, 0.089)	0.874	0.230	0.015
	sO_2_	0.0051 (-0.034, 0.044)	0.738	0.340	0.004
	ΔsO_2_	−0.0014 (-0.036, 0.033)	0.216	0.692	0.003
2	HbT %BP Hb	0.019 (-0.085, 0.12) −0.162 (-1.79, 1.47) 0.0094 (-0.043, 0.062)	0.841 0.612 0.837	0.262 0.428 0.265	0.030 7 0.008
	HbO_2_	0.0094 (-0.042, 0.060)	0.846	0.256	0.007
	sO_2_	0.0018 (-0.0055, 0.0092)	0.911	0.188	<0.001
	ΔsO_2_	−0.0013 (-0.0027, 9.34e-05)	0.993	0.057	5.79e-06
3	HbT %BP Hb	0.022 (-0.10, 0.15) 0.0071 (-0.490, 0.504) 0.012 (-0.079, 0.10)	0.829 0.031 0.742	0.271 0.887 0.339	0.043 0.688 0.023
	HbO_2_	0.0096 (-0.025, 0.044)	0.927	0.174	0.003
	sO_2_	0.00041 (-0.010, 0.011)	0.189	0.018	<0.001
	ΔsO_2_	0.0013 (-0.0067, 0.0093)	0.804	0.291	1.86e-04
4	HbT %BP	0.0084 (-0.20, 0.22) 0.204 (-19.3, 19.7)	0.207 0.017	0.700 0.917	0.122 1062
	Hb	0.0034 (-0.11, 0.11)	0.136	0.762	0.033
	HbO_2_	0.0050 (-0.096, 0.11)	0.289	0.639	0.028
	sO_2_	0.0031 (-0.090, 0.096)	0.153	0.155	0.024
	ΔsO_2_	-0.00024 (-0.011, 0.011)	0.071	0.829	3.40e-04

No significant differences were found between the (temporal) signal change calculated for the repositioning study and that obtained for the longitudinal (75 min) study, for ROI averaged Hb, HbO_2_, HbT and sO_2_, apart for the HbT parameter for animal 2. Figs. S3–S5 show the longitudinal and re-positioning data for Hb, HbO_2_, HbT and SO_2_, for each animal, plotted in the same figure, and the p-values showing the similarity of the slopes, i.e. if pvalue<0.05 the temporal change in signal was significantly different between the 75 min and re-positioning studies.

The slice- and intra-tumour CoVs for ROI-averaged Hb, HbO_2_, HbT and sO_2_, are summarised in Table S2. The intra-tumour CoV measurement of animal four, excluding position 2, where 46% of signal was lost, is also shown. As was seen for the 75-minute study, the intra-tumour CoVs for measurements acquired after re-positioning the animals was higher for the haemoglobin components (Hb: 22.0±6.0%; HbO_2_: 40.0±11.0%; HbT: 28.1±5.1%) than for blood sO_2_ (22.0 ± 21.2% and 15.0 ± 11.3% with and without considering position 2 imaging for animal 4, respectively). As for the ΔsO_2_ value, the intra-CoV was of 30.3 ± 11.3%. The slice-CoV was also lower (19.4 ± 18.4% including animal 4, position 2 imaging) than the intra-tumour CoV.

The inter-CoV calculated was 25.8±16.4% for the average Hb, 45.6±19.6% for the average HbO_2_, 30.6±15.6% for the average HbT and 26.5±8.3% for the average sO_2_. Excluding that data point, the inter-CoV calculated was of 20.4±16.4% for the average Hb, 33.2±6.4% for the average HbO_2_, 22.7±6.8% for the average HbT and 13.0±13.2% for the average sO_2_. The inter-CoV for ΔsO_2_ value was high (57.3 ± 18.6%).

### 3.2 Variation during tumour growth, 6 day study

For the long-term study, 10 CAL^R^ tumours were imaged. The ROI-averaged Hb, HbO_2_, HbT, blood sO_2_ and ΔsO_2_ from 3 central slices per tumour, measured during air-breathing and oxygen-breathing, for the long term (6 day) variability study (total of 4 imaging sessions) are shown in Figs. S7–S9.

From this data, the inter- and intra-tumour CoV for air-breathing photoacoustic imaging, for 4 imaging sessions acquired over 6 days, were calculated and are summarised in Tables S4 and S5. As was found for the short term variability studies, the intra- and inter-tumour CoVs for the sO_2_ parameter were lower (7.5±2.5% and 13.1±3.2%, respectively) than for the haemoglobin and ΔsO_2_ parameters (range 19.3±8.7%–39.7±5.6%).

### 3.3 Tumour growth rate

**Fig. 6:**
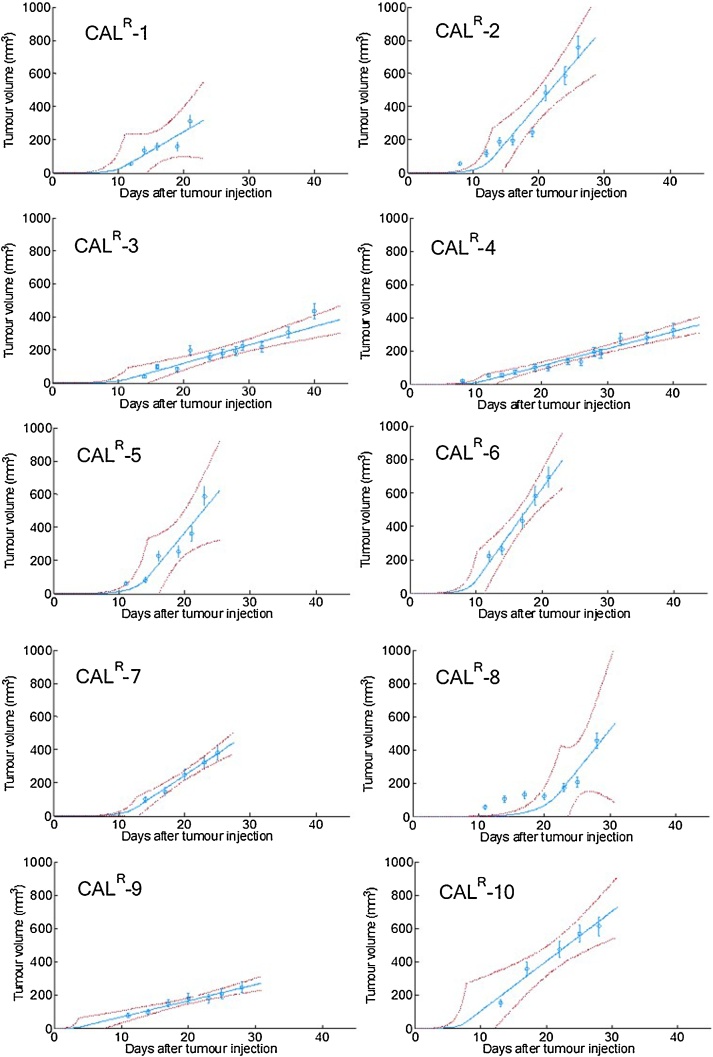
CAL^R^ tumour volume, as measured by calipers (blue circles). Error bars represent tumour volume error, as described by Eq. 2. The exponential-linear fitted model is shown by the blue full line with 95% confidence intervals (dashed red lines).

Growth rates calculated for ten CAL^R^ tumours used in the longitudinal study are shown in Table 3. The individual fitted growth curves, as well as the exponential-linear model fitting, are shown in Fig. 6. The uncertainties (95% confidence intervals) are large and hence no statistically significant differences were found between individual tumour growth rates. The goodness of fit, R^2^, for the majority of the tumours (N = 8) was ≥ 0.88, suggesting good agreement between the exponential-linear model and the data. Animals CAL^R^-1 and CAL^R^-8, had abnormally low fits to the model. CAL^R^-1 ulcerated, which can affect the tumour growth (see Fig. 6). Fig. 6 shows stagnated tumour growth (108 ± 18 mm^3^ to 123 ± 20 mm^3^) between days 12–20 after tumour implantation for CAL^R^-8.

**Table 3:** Exponential (α_0_) and linear (α_1_) growth rates, and time after implantation for transition between exponential and linear phase, τ, for 10 CAL^R^ tumours, including the goodness of fit (R^2^) of the exponential-linear model to the growth curves.

Mouse	α0 (day^−1^)	95% confidence interval for α_0_	α1 (mm^3^.day^−1^)	95% confidence interval for α_1_	τ (days after tumour implant)	Goodness of fit (R^2^)
CAL^R^-1	0.55	[0.24; 0.85]	23	[0.48; 45]	5	0.77
CAL^R^-2	0.53	[0.41; 0.65]	46	[29; 63]	6	0.92
CAL^R^-3	0.48	[0.28; 0.68]	11	[8; 14]	5	0.90
CAL^R^-4	0.48	[0.36; 0.60]	10	[9; 12]	5	0.95
CAL^R^-5	0.48	[0.36; 0.60]	48	[16; 79]	6	0.88
CAL^R^-6	0.66	[0.52; 0.81]	55	[38; 72]	4	0.97
CAL^R^-7	0.50	[0.43; 0.56]	26	[21; 32]	5	0.99
CAL^R^-8	0.33	[0.26; 0.39]	49	[0; 110]	10	0.64
CAL^R^-9	1.2	[0; 2.7]	10	[7; 12]	2	0.95
CAL^R^-10	0.77	[0.22; 1.3]	30	[18; 42]	3	0.96

**Fig. 7:**
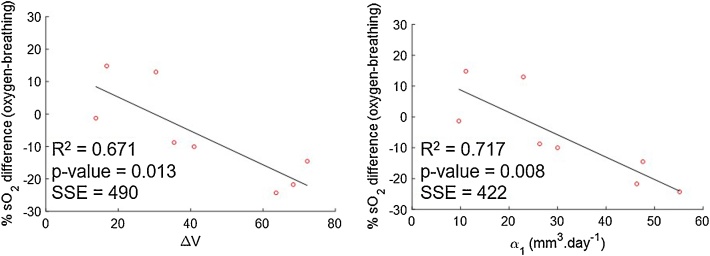
Correlation between rate of change in volume between day 1 and 6 of imaging, ΔV, or linear growth rate, α1, and the percentage difference in sO_2_ between days 1 and 6 of imaging for 8 CAL^R^ tumours, for oxygen-breathing measurements.

Table 3 shows that the time at which tumour growth changed from exponential to linear varied from 2 to 10 days after tumour implant. Based on these results and the growth curves shown in Fig. 6, all photoacoustic imaging acquired for this study was performed during the linear growth phase. In order to investigate whether these changes were related to change in volume over time (ΔV), calculated using raw caliper volume measurements, or linear rate of tumour growth (α_1_), estimated using the exponential-linear model, the relationship between these parameters and the percentage difference in sO_2_ between day 6 and day 1 was investigated and is shown in Fig. 7. A good negative correlation between the percentage difference in sO_2_ and both ΔV and α_1_, is seen, during oxygen-breathing, i.e. the larger the decrease in sO_2_ (oxygen-breathing), the faster the tumour was growing. Air-breathing data is shown in Fig. S10.

## 4 Discussion

The main objective of this paper was to explore the variation of the blood sO_2_, HbO_2_, Hb and HbT parameters in untreated subcutaneous head and neck tumours measured with a tomographic photoacoustic system, with time, over short (minutes-hours) and long periods (several days), and with position of the animals in the imaging cradle. From these studies, it was possible to quantify the variability due to anaesthesia, tumour growth and re-positioning. This baseline information is required for assessing the significance of any changes in these characteristics that may be related to response to treatment, in future studies of the effects of radiotherapy and/or HIFU.

Initially, short-term variability of the PAI parameters was assessed. The photoacoustic signal variability over 75 min was measured to investigate whether the injectable anaesthesia could affect haemoglobin and blood sO_2_ measured in tumours. Fig. 2 shows a decreasing trend in black pixels, regions where HbT is too low to be measured, after the first 10 min of imaging. Shah et al. [23] showed, by co-registration of PAI (using this system) and microbubble contrast-enhanced ultrasound images, that the black regions in tumours (in mice) did not lack perfusion, although they typically exhibited lower levels of perfusion and later microbubble arrival than other regions within the tumour. The MSOT and the software available at the moment are still 1st generation and efforts are being made to improve the detection of HbT in order to eliminate the occurrence of black pixels.

During the 75 min in which in the percentage of black pixels tended to decrease, an increase in Hb, HbO_2_ and HbT (Fig. 2 and Table 1) was measured, resulting in increased blood sO_2_ for 3 out of 4 animals. These results suggest peripheral vasodilation. The combination of medetomidine, fentanyl and midazolam used for this study has been shown to cause a significant increase in blood pressure in rabbits, rats, and mice, which could arise from an increase in vascular resistance or peripheral vasoconstriction [33,37,38]. However, it is well-known that the cardiovascular effects of the combination of these anaesthetic agents vary in different species [39] and to the authors’ knowledge, there have been no reports on its effect in female FOXnu^n1^ mice. Furthermore, there is a trend for decrease in sO2 and decrease in body temperature. Our results suggest that these are not the cardiovascular effects we are measuring. The increase in HbT and sO_2_ in our study can be due to a cardiovascular effect of the anaesthetic agent combination, due to an influence of the hydrodynamic pressure when the animal is enveloped in a plastic film and submerged inside the water tank, or due to an influence of the compression of the tumour due to the animal’s body weight, which possibly stops blood outflow from the tumour. All these factors could have contributed to the trend that was observed and further work is necessary to understand its source. Indeed, a study of the effects of the MSOT experimental setup on the animal’s tissue and blood gases would be quite important, but it is not possible to perform with the current hardware.

The trend for an increase in haemoglobin was observed during the whole duration of imaging session, so the signal does not stabilise during an acceptable period of anaesthesia for a small animal. Therefore, this baseline change in signal needs to be considered in future studies, as it is not possible to wait for long enough for a stable signal to be seen before starting imaging the animal.

The inter- and intra-tumour CoVs (as shown in Table S1) were similar in this study, >16.0 ± 8.4% and >20.9 ± 3.1%, respectively, for the parameters analysed: Hb, HbO_2_, HbT and sO_2_. This reflects a high variation in the measured signal of each animal, probably due to the increasing trend in haemoglobin and blood sO_2_ over time. Joseph et al. (2017) [30] studied the repeatability and reproducibility of PAI with the same system as that used here in the left kidney and spleen of 8–12 week old mice (n = 7), acquiring images over a period of 90 min, under isoflurane anaesthesia. Interestingly, with this anaesthetic, the authors found a steady increasing trend in HbT and blood sO_2_ over the 90 min imaging acquisition time. The group reported that the intra-organ CoVs for haemoglobin (Hb, HbO_2_ and HbT, measurements averaged over a single ROI) for spleen was below 16.4 and for kidney below 18.5, while for sO_2_ these values dropped to 3.9 and 3.4, respectively. The CoVs measured in this paper during 75 min of imaging in subcutaneous tumours were higher than those reported in spleen and kidney, although it is not possible to compare the uncertainties in the measurements. In the experiment conducted by Joseph et al [30], the organs (spleen and left kidney) were located deep within the animal’s body, so there was likely to be minimal compression of their blood supply due to mounting in the system, unlike the situation for subcutaneous tumour, located superficially. This is in addition to the deficient vascular supply that renders tumours prone to ‘cyclic’ hypoxia [2], which can affect the measurements of haemoglobin and oxygen saturation.

In the second study reported here, the effects of re-positioning the animal three times during one imaging session were analysed. Fig. 4 shows that the shape of the tumour may vary considerably during repositioning of the animal, changing the size of the tumour-ROI used for analysis. Subcutaneous tumours, besides being subject to compression due to the animal’s body weight acting against buoyancy, as well as due to hydrostatic pressure, can move relative to underlying anatomy as the skin can be highly mobile. Therefore, during longitudinal studies it would be important for the user to place the animal and tumour in the same position relative to the MSOT mouse holder. From Fig. 4, it is possible to observe differences in the ‘oxymaps’ depending on the position of the animal, which can result in differences in the distribution and intensity of blue/red pixels. A radial gradient in blood sO_2_ was observed for all animals (as well as in the 75 min study, Fig. 3). This might be due to lack of light fluence correction, ie, the MSOT system does not take in consideration changes in light absorption with depth, although efforts are being made to implement this correction [39]. In mouse 4, position 2 resulted in an atypically high number of black pixels (46%), with correspondingly uncharacteristically low levels of Hb, HbT and sO_2_. However, this was observed in only one imaging scan, for one animal out of twelve, so the likelihood of this type of artefact is very low.

In order to investigate whether the differences in ‘oxymaps’ during the re-positioning study also resulted in significant differences in ROI-averaged photoacoustic imaging measurements (Hb, HbO_2_, HbT and sO_2_) over time, the temporal changes in these measurements were calculated. A trend for an increase in the haemoglobin components amount was also observed, as in the 75-minute study. Animal 3 exhibited no trend in levels of sO_2_ compared to the other two animals, consistent with the observations of the sequential imaging for a period of 75 min. Table 2 shows that for this study, the p-values were >0.05 so the slopes (change in signal) were not significantly different from zero. This is likely to be due to the low (n = 3 positions) number of points, per animal.

From Figs. S3–S5, it is also possible to observe that there were no significant differences between the various positions, for the slopes representing the variation over the 75 min of the imaging study. The imaging for animal 4, in position 2 that resulted in a large number of black pixels, increased the inter- and intra-tumour CoV for the re-positioning study. However, the majority of the signal in this imaging scan was lost, so it is not possible to know if this decrease is a result of changes in the tumour or simply an artefact due to the signal loss.

The inter- and intra-tumour CoV for the CAL^R^ tumours due the re-positioning of the animal (Table S2) were >22.0 ± 21.2% and >26.5 ± 8.3%, respectively, for all the parameters (Hb, HbO_2_, HbT and sO_2_), and hence similar to those obtained for the study without re-positioning the animal (see Table S1), over a 75-minute period, apart from animal 4. This also suggests that anaesthesia is the main experimental factor that contributes for the temporal variability of the signal, as found by Joseph et al. [30].

The intra- and inter-tumour CoVs for the 6 day imaging study were >19.3 ± 8.7% and >27.0 ± 4.1%, respectively, for the haemoglobin parameters (Hb, HbO_2_ and HbT). The sO_2_ parameter showed a lower intra-tumour CoV (7.5 ± 2.5%) in the long term studies, in comparison to Hb, HbO_2_ and HbT. Joseph et al. [30] also demonstrated, by imaging the animals on 3 consecutive days, that while the sO_2_ was constant over time in kidney (maintained between 0.8 and 0.7) and spleen (maintained between 0.6 and 0.8), for HbT the variation was larger (between 25–16 A.U and 9–13 A.U. for spleen and left kidney, respectively). These studies suggest that variation in blood sO_2_ is expected to be lower than that for haemoglobin. Also, the intra-tumour blood sO_2_ variability is lower for the long-term study, over 6 days, compared to that obtained for the 75 min (16.0 ± 8.4%) and repositioning (22.0 ± 21.2%) studies, once more showing that time under anaesthesia per imaging session should be minimised. The ΔsO_2_ CoVs were high (>30%) in both the 6-day and repositioning study, hence we did not consider this parameter to be as reliable as in Joseph et al. [30] study.

In order to assess whether there was any dependence of tumour blood sO_2_ on growth, the growth rates of the 10 CAL^R^ tumours during two different growth stages, were calculated using the exponential-linear model. The goodness of fit showed a good correlation (R^2^>0.8) between the model and 8 datasets. Of the two animals omitted from analysis, one of the tumours became ulcerated, which is known to introduce an error in tumour measurement [41] and the other animal showed an unexpected delay in tumour growth. Fig. 7 shows a good linear relationship between the linear growth rate and the variation in sO_2_ (R^2^ = 0.72, n = 8), during oxygen-breathing. A negative linear relationship was found between the rate of change in volume calculated just for the 6 days of imaging and percentage change in sO_2_ (R^2^ = 0.67). Tumours that grew faster (n = 5) during the linear phase (α1 > 20 mm.day^−1^) had a decrease in sO_2_ [range of −10% to −56%] between days 1 and 6 of imaging, probably because they outgrew their blood supply before new vasculature could be formed. Slower growing tumours (n = 3) exhibited an increase or no change in sO_2_ [+12% and -1%], possibly because angiogenesis was occurring at a rate similar to the tumour growth rate. Interestingly, Tomaszewski et al. [40] used oxygen enhanced (OE)-PAI, to visualise the spatiotemporal heterogeneity of tumour vascular function in a poorly differentiated and aggressive prostate tumour model, PC3. They showed that sO_2_ (while O_2_ breathing) and ΔsO_2_ also decreased with tumour growth, from a 30 mm^3^ volume to time of culling. The CAL^R^ model used for this project has also been shown to develop rapidly and become hypoxic [40]. This observation showed that tumours of the same cell-line, although inoculated on the same day in the same-mouse strain, grow differently and this inherent biological variability should be considered for studies involving long term assessment of vasculature changes in response to a therapy.

A limitation of this study is that it evaluated the baseline variations in the photoacoustic signal for only one tumour type, a subcutaneous head and neck tumour model. Other tumour types might show different variation. For example, Tomaszewski et al. [36] showed that two prostate tumour models, PC3 and LNCaP, exhibited different haemodynamic behaviour, as assessed by PAI.

## 5 Conclusions

This paper documents the variation in haemoglobin (Hb, HbO_2_ and HbT) and oxygen saturation over short (up to 75 min) and long (6 days) periods, and the variation with repositioning of the animal, in untreated subcutaneous head and neck tumours in nude mice. Time under anaesthesia is the largest source of temporal variability in the photoacoustic signal. To perform inter- and intra-animal comparison in longitudinal studies, for example when assessing the effects of treatment, ideally the imaging should be started at a fixed time point after anaesthesia and, if possible, sham-treated animals should be imaged at the same time points as the treated animals.

Positioning of the animal was found to influence image quality in a limited number of animals; when reconstruction artefacts or an unexpected absence in PAI signal are observed during the preview of the scan, the animal can be removed and re-positioned to obtain improved PAI signal. With a carefully planned experimental protocol, photoacoustic imaging could be used for assessing changes in tumour haemoglobin and oxygenation in longitudinal studies, including those designed to assess cancer therapies that may affect vasculature and, consequently, tumour oxygenation.

## Data availability

All data and/or analysis performed in this study are available from the corresponding author on request.

## Conflict of interest

The authors declare that there are no conflict of interest.
