# Supplementary material for: Quantitative photoacoustic imaging study of tumours in vivo: Baseline variations in quantitative measurements
Source: Photoacoustics. 2018 Dec 7;13:53–65. doi: 10.1016/j.pacs.2018.12.002 (PMC6297191; doi:10.1016/j.pacs.2018.12.002)
Supplement: Supplementary file 1 [file mmc1.pdf]

## Supplementary material

### Supplementary Methods

#### Tumour growth rates calculation

Tumour growth curves were fitted to the Exponential-Linear Model to calculate tumours' growth rates. The model assumes that cells initially proliferate with constant cell cycle duration, $T_c$, resulting in exponential growth, followed by a linear growth phase, when there is a decrease in actively proliferating cells and these are constrained to the margins of the tumour. The model is described as follows:

$$\frac{\partial V}{\partial t} = \alpha_0 V, t \leq \tau$$

$$\frac{\partial V}{\partial t} = \alpha_1, t > \tau$$

Eq. S1

where V is tumour volume ($mm^3$), t is time (days), $\alpha_0$ is the growth rate during the exponential phase, i.e. the fraction of proliferating cells at time $\ln 2/T_c$, $\alpha_1$ is the growth rate in the linear phase and $\tau$ represents the time point at which the growth changes from the exponential to the linear phase. Assuming the solution is continuously differentiable then:

$$\tau = \frac{1}{\alpha_0} \log(\frac{\alpha_1}{\alpha_0 V_0})$$

Eq. S2

where $V_0$ is the initial volume, in this case 0.1 $mm^3$, chosen as an arbitrary small initial volume of tumour cells.

The tumour growth fitting was carried out in MATLAB (2013a, 8.1.0.604), using a nonlinear least squared fit to obtain the $\alpha_0$ and $\alpha_1$ parameters. Fits were performed on volume data weighted using the measurement uncertainties of each data point, which corresponded to the Verror calculated from Eq. 2, within 95% confidence interval bounds.

## Supplementary Tables

**Table S1:** Summary of slice- and intra-tumour CoVs of the measurements of each animal acquired over 75 minutes, and the average Hb, $HbO_2$, HbT and $sO_2$ measured during that time period.

| Animal | ROI-averaged Parameter | average±std | Intra-tumour CoV (%) | Average slice-CoV (%) |
|---|---|---|---|---|
| 1 | Hb | 0.99±0.30 | 30.1 | 6.4±2.6 |
| | $HbO_2$ | 0.71±0.36 | 51.5 | 5.5±1.9 |
| | HbT | 1.72±0.68 | 39.3 | 4.2±2.2 |
| | $sO_2$ | 0.40±0.07 | 18.7 | 5.0±2.1 |
| 2 | Hb | 1.12±0.29 | 25.5 | 2.4±0.8 |
| | $HbO_2$ | 0.66±0.31 | 47.1 | 6.5±5.4 |
| | HbT | 1.78±0.60 | 33.5 | 1.5±1.2 |
| | $sO_2$ | 0.35±0.06 | 18.0 | 5.6±4.2 |
| 3 | Hb | 1.00±0.18 | 18.1 | 2.5±1.2 |
| | $HbO_2$ | 0.80±0.16 | 20.3 | 4.0±2.4 |
| | HbT | 1.81±0.34 | 19.0 | 1.7±0.6 |
| | $sO_2$ | 0.44±0.01 | 2.2 | 3.3±1.5 |
| 4 | Hb | 0.67±0.11 | 16.2 | 10.9±2.6 |
| | $HbO_2$ | 0.27±0.11 | 41.9 | 13.9±7.4 |
| | HbT | 0.94±0.23 | 23.9 | 9.3±2.5 |
| | $sO_2$ | 0.28±0.07 | 24.9 | 11.5±7.4 |
| Average intra-tumour CoV (n=4) | Hb | 22.5±6.5 | | |
| | $HbO_2$ | 40.2±13.9 | | |
| | HbT | 22.9±9.2 | | |
| | $sO_2$ | 16.0±8.4 | | |

**Table S2:** Summary of average slice- and intra-tumour CoV of the measurements after re-positioning each animal 3 times, during air-breathing imaging, and the average Hb, $HbO_2$, HbT and $sO_2$.

| Animal | Parameter | average (±std) | Intra- tumour CoV (%) | Average Slice-CoV (%) |
|---|---|---|---|---|
| 1 | Hb | 0.86±0.21 | 24.3 | 2.6±1.2 |
| | $HbO_2$ | 0.46±0.24 | 52.7 | 6.9±2.1 |
| | HbT | 1.32±0.45 | 34.0 | 2.9±1.0 |
| | $sO_2$ | 0.33±0.09 | 27.3 | 5.3±2.9 |
| 2 | Hb | 0.62±0.15 | 24.8 | 3.7±3.4 |
| | $HbO_2$ | 0.40±0.15 | 38.1 | 7.1±3.6 |
| | HbT | 1.02±0.31 | 30.0 | 3.7±2.1 |
| | $sO_2$ | 0.39±0.03 | 7.5 | 4.9±3.2 |
| 3 | Hb | 0.82±0.21 | 26.0 | 4.7±3.2 |
| | $HbO_2$ | 0.57±0.15 | 26.4 | 7.1±3.9 |
| | HbT | 1.38±0.36 | 25.9 | 2.8±1.7 |
| | $sO_2$ | 0.41±0.01 | 3.4 | 6.3±3.8 |
| 4 | Hb | 0.47±0.14 | 29.1 | 5.6±0.8 |
| | $HbO_2$ | 0.18±0.14 | 78.3 | 19.4±18.4 |
| | HbT | 0.65±0.28 | 42.5 | 6.5±3.1 |
| | $sO_2$ | 0.24±0.12 | 49.7 | 15.4±15.8 |
| 4 (excluding position 2) | Hb | 0.55±0.07 | 13.1 | 5.1±0.2 |
| | $HbO_2$ | 0.25±0.11 | 42.9 | 9.0±5.8 |
| | HbT | 0.80±0.18 | 22.4 | 5.1±2.8 |
| | $sO_2$ | 0.30±0.07 | 21.7 | 6.3±1.3 |
| Average intra-tumour CoV (n=4) | Hb | 26.0±2.2 | | |
| | $HbO_2$ | 48.9±22.4 | | |
| | HbT | 33.1±7.1 | | |
| | $sO_2$ | 22.0±21.2 | | |
| Average intra-tumour CoV (without position 2, mouse 4) | Hb | 22.0±6.0 | | |
| | $HbO_2$ | 40.0±11.0 | | |
| | HbT | 28.1±5.1 | | |
| | $sO_2$ | 15.0±11.3 | | |

**Table S3:** Rate of change of ROI-averaged Hb, $HbO_2$, HbT, $sO_2$ and percentage of black pixels (%BP) signal during the re-positioning study (oxygen-breathing). The goodness of fit, $R^2$, indicates how much of the signal variability is time dependent and the p-value if the signal change (slope of the linear model) is significantly different from zero. The SSE reflects how similar the predicted values and measured data are, i.e. the closest SSE is to zero, the better the linear fit.

| Animal | | Signal change, $min^{-1}$ (95% CI) | $R^2$ | p-value | SSE |
|---|---|---|---|---|---|
| 1 | Hb | 0.015 (-0.033, 0.062) | 0.939 | 0.158 | 0.006 |
| | $HbO_2$ | 0.023 (-0.022, 0.069) | 0.977 | 0.098 | 0.006 |
| | HbT | 0.038 (-0.055, 0.13) | 0.964 | 0.121 | 0.024 |
| | $sO_2$ | 0.0032 (-0.0049, 0.011) | 0.962 | 0.126 | <0.001 |
| | %BP | -0.858 (-5.39, 3.67) | 0.853 | 0.251 | 57 |
| 2 | Hb | 0.0089 (-0.060, 0.077) | 0.732 | 0.348 | 0.013 |
| | $HbO_2$ | 0.0093 (-0.059, 0.078) | 0.752 | 0.333 | 0.013 |
| | HbT | 0.018 (-0.12, 0.15) | 0.742 | 0.339 | 0.051 |
| | $sO_2$ | 0.0006 (-0.0021, 0.0032) | 0.883 | 0.228 | <0.001 |
| | %BP | -0.280 (-2.70, 2.14) | 0.685 | 0.379 | 16 |
| 3 | Hb | 0.014 (-0.070, 0.098) | 0.817 | 0.281 | 0.020 |
| | $HbO_2$ | 0.017 (-0.023, 0.057) | 0.965 | 0.120 | 0.004 |
| | HbT | 0.031 (-0.093, 0.15) | 0.908 | 0.197 | 0.043 |
| | $sO_2$ | 0.0018 (-0.0044, 0.0079) | 0.932 | 0.160 | <0.001 |
| | %BP | 0.0038 (-0.194, 0.202) | 0.056 | 0.848 | 0.109 |
| 4 | Hb | 0.0062 (-0.094, 0.11) | 0.380 | 0.579 | 0.028 |
| | $HbO_2$ | 0.0071 (-0.12, 0.13) | 0.346 | 0.601 | 0.043 |
| | HbT | 0.0084 (-0.20, 0.22) | 0.208 | 0.589 | 0.141 |
| | $sO_2$ | 0.0027 (-0.10, 0.11) | 0.101 | 0.793 | 0.030 |
| | %BP | -0.349 (-19.1, 18.2) | 0.053 | 0.852 | 979 |

**Table S4:** Summary of inter- and intra-tumour CoVs for the $CAL^R$ tumours (N=10), over a 6 day period, for ROI-averaged Hb, $HbO_2$, HbT and $sO_2$, during air-breathing imaging. $\Delta sO_2$ is also included in this table.

| Parameter | Intra-tumour CoV (%) | Inter-tumour CoV (%) |
|---|---|---|
| Hb | 19.3±8.7 | 27.0±4.1 |
| $HbO_2$ | 22.8±15.4 | 39.7±5.6 |
| HbT | 20.6±12.0 | 32.7±4.7 |
| $sO_2$ | 7.5±2.5 | 13.1±3.2 |
| $\Delta sO_2$ | 63.5±45.7 | 74.0±11.5 |

**Table S5:** Summary of inter- and intra-tumour CoVs for the $CAL^R$ tumours (N=10), over a 6 day period, for ROI-averaged Hb, $HbO_2$, HbT and $sO_2$, during oxygen-breathing imaging.

| Parameter | Intra-tumour CoV (%) | Inter-tumour CoV (%) |
|---|---|---|
| Hb | 41.2±15.2 | 29.8±5.6 |
| $HbO_2$ | 19.5±11.7 | 35.9±4.5 |
| HbT | 22.4±11.7 | 31.3±4.4 |
| $sO_2$ | 6.5±3.2 | 11.8±3.6 |

## Supplementary Figures

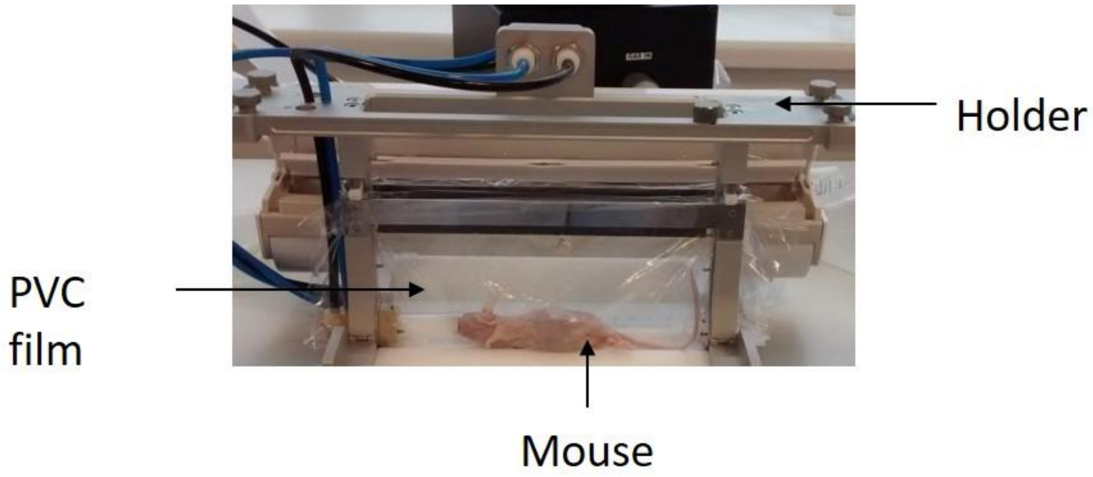


**Fig. S1:** Photograph showing the experimental setup of the mouse, in the MSOT holder prior to mounting in the imaging system.

**Fig. S2:** ROI-averaged percentage of black pixels, and Hb, $HbO_2$, HbT and $sO_2$ signals, for $CAL^R$ tumours (n=4) imaged after being removed and re-positioned in the tank three times, during oxygen-breathing. Error bars represent the standard deviation over 3 adjacent central tumour slices, 1 mm apart. Solid lines represent a linear regression fitted to the data.

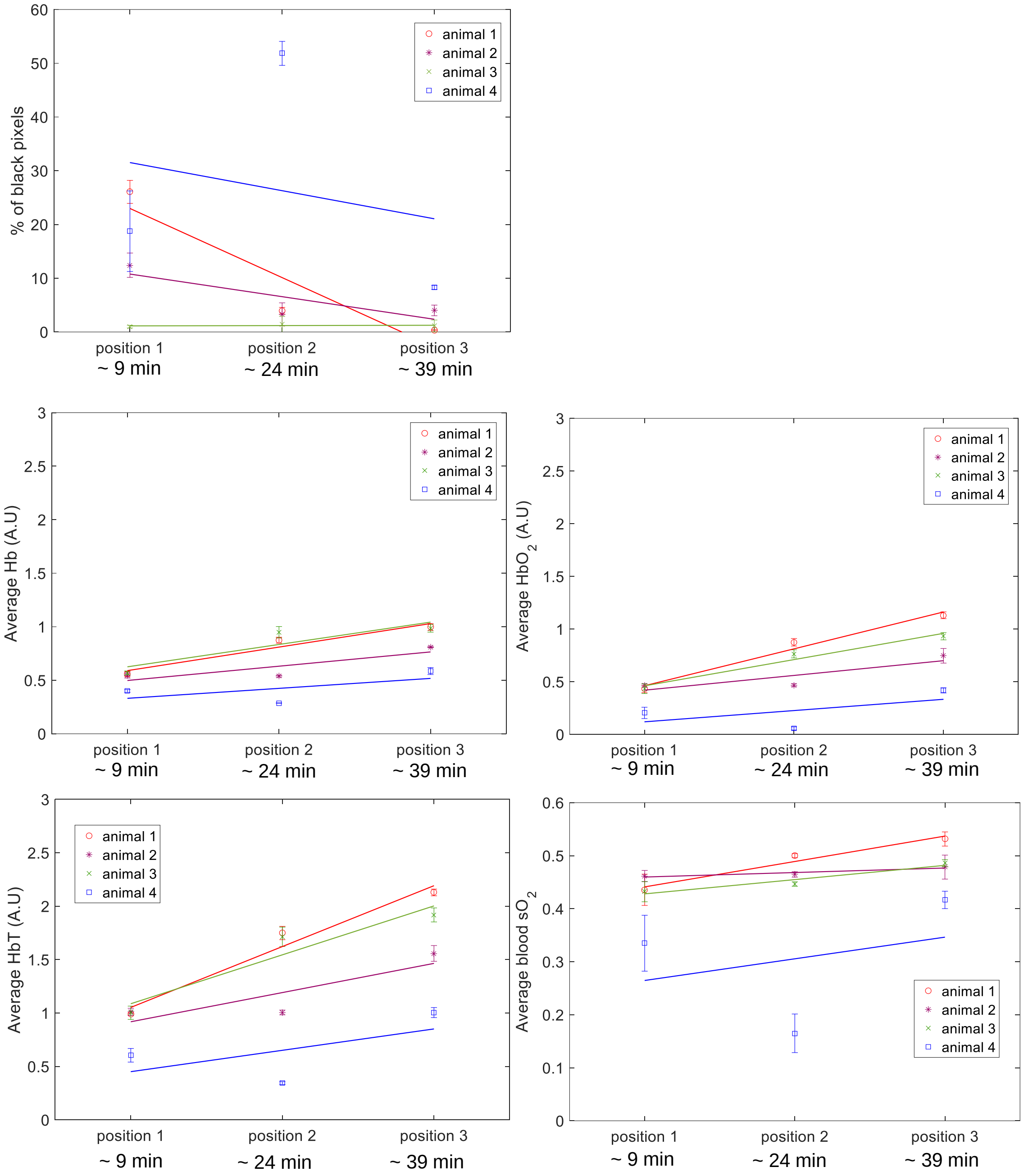


**Fig. S3:** Comparison of Hb change between longitudinal (75 minute) and re-positioning studies. p-values indicate if the differences between the two slopes resultant from a linear model fitting are statistically significant (if p-value<0.05).

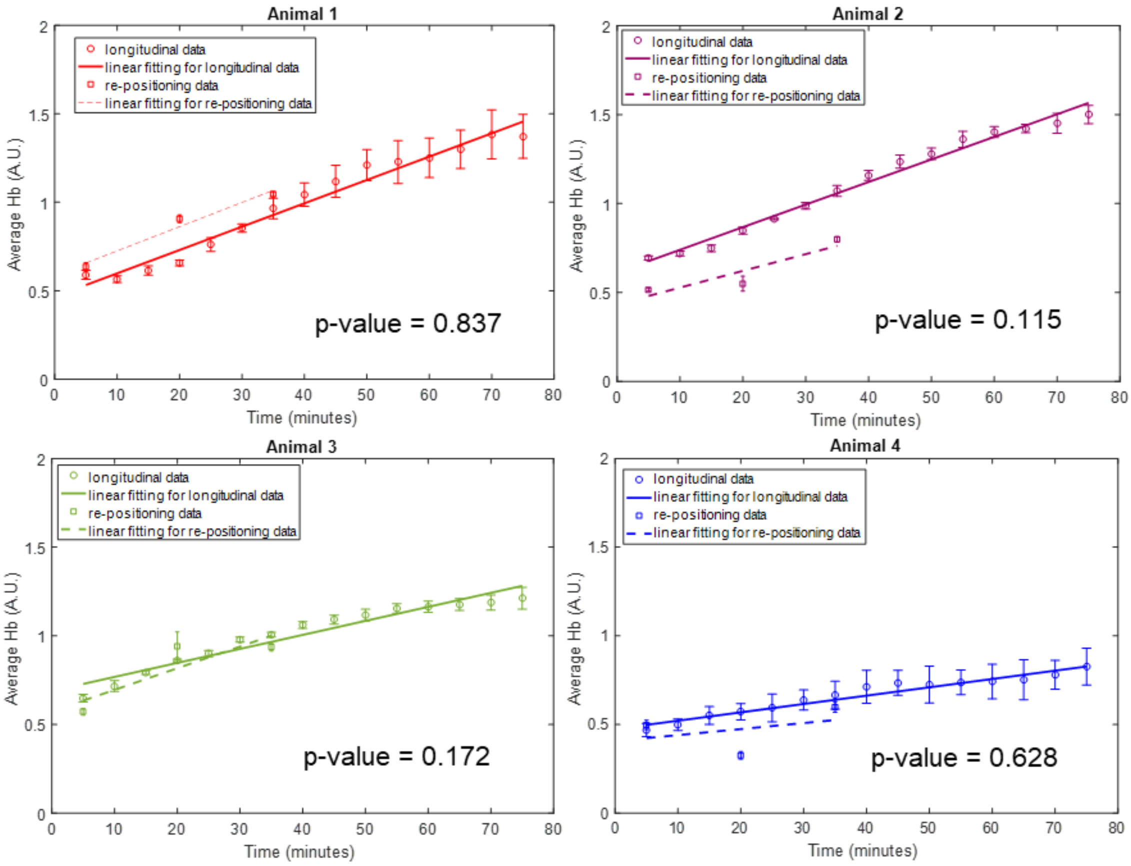
Animal 1
longitudinal data
linear fitting for longitudinal data
re-positioning data
linear fitting for re-positioning data
p-value = 0.837
Average Hb (A.U.)
Time (minutes)
Animal 2
longitudinal data
linear fitting for longitudinal data
re-positioning data
linear fitting for re-positioning data
p-value = 0.115
Average Hb (A.U.)
Time (minutes)
Animal 3
longitudinal data
linear fitting for longitudinal data
re-positioning data
linear fitting for re-positioning data
p-value = 0.172
Average Hb (A.U.)
Time (minutes)
Animal 4
longitudinal data
linear fitting for longitudinal data
re-positioning data
linear fitting for re-positioning data
p-value = 0.628
Average Hb (A.U.)
Time (minutes)

**Fig. S4:** Comparison of $HbO_2$ change between longitudinal (75 minute) and re-positioning studies. p-values indicate if the differences between the two slopes resultant from a linear model fitting are statistically significant (if p-value<0.05).

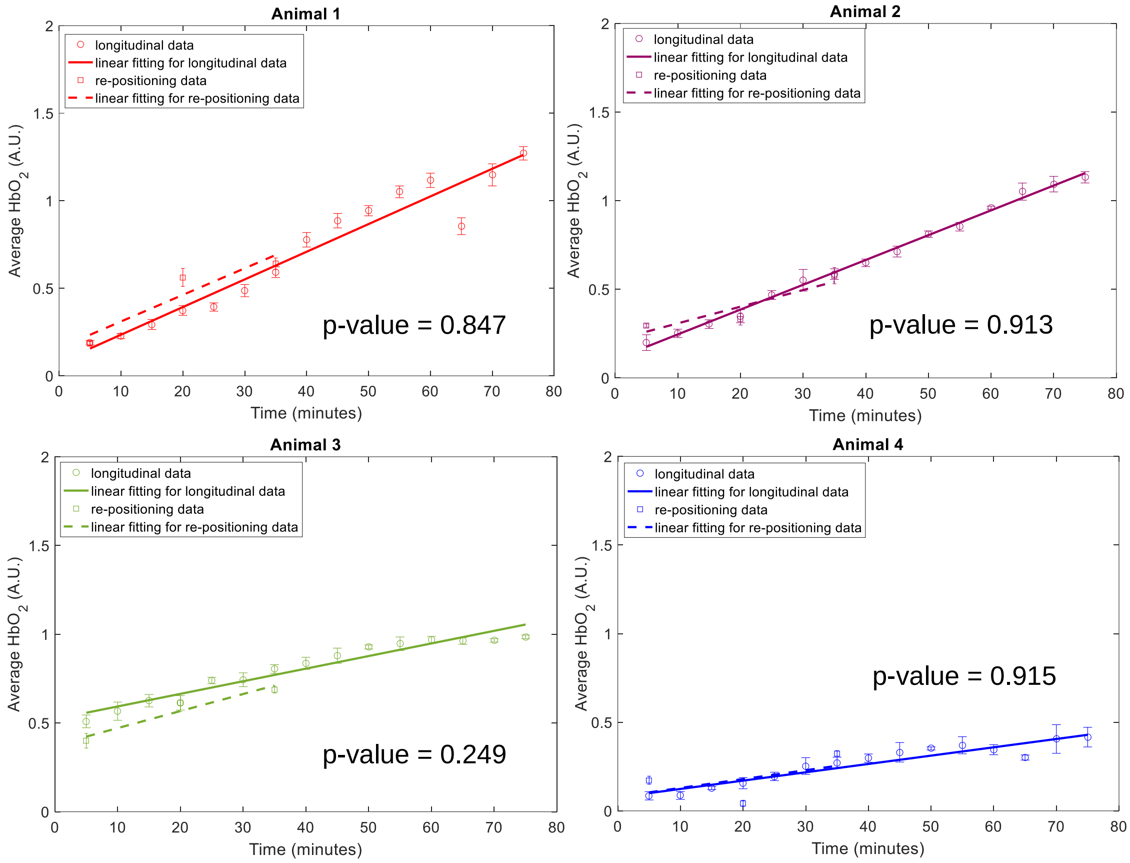

**Fig. S5:** Comparison of HbT change between longitudinal (75 minute) and re-positioning studies. p-values indicate if the differences between the two slopes resultant from a linear model fitting are statistically significant (if p-value<0.05).

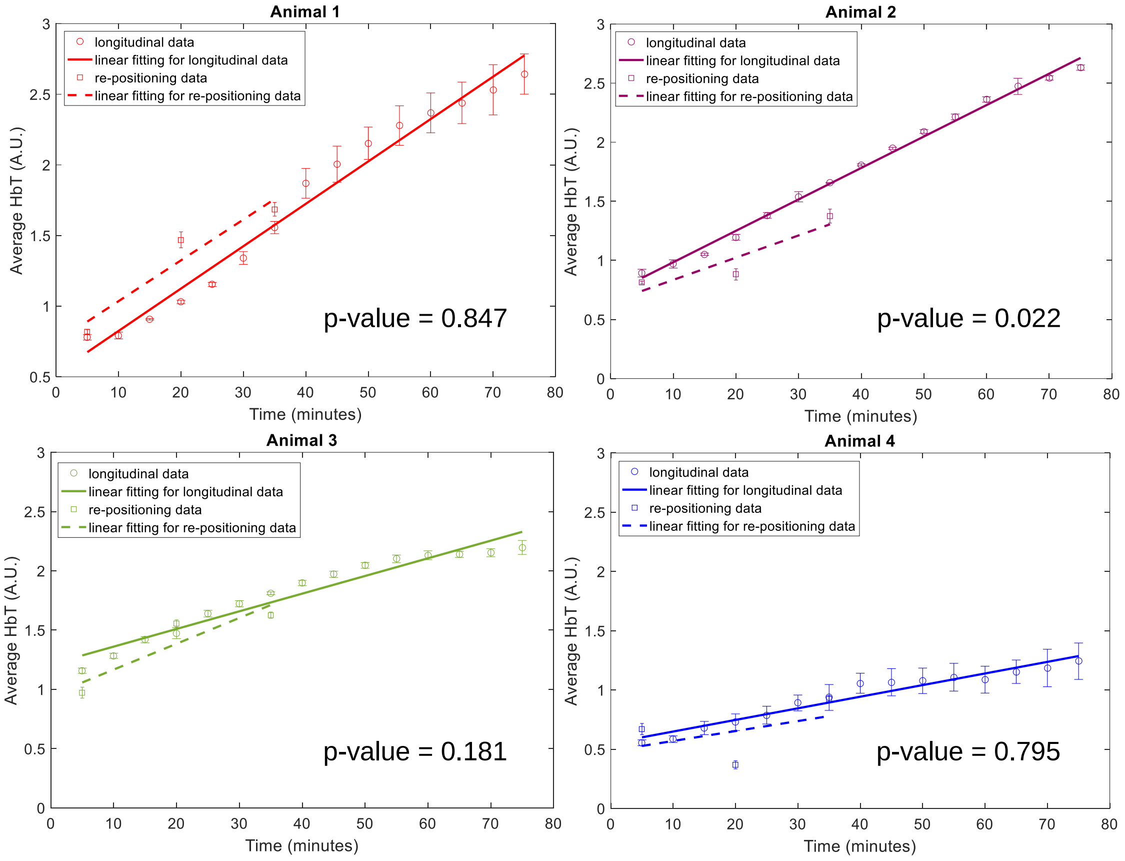

**Fig. S6:** Comparison of $sO_2$ change between longitudinal (75 minute) and re-positioning studies. p-values indicate if the differences between the two slopes resultant from a linear model fitting are statistically significant (if p-value<0.05).

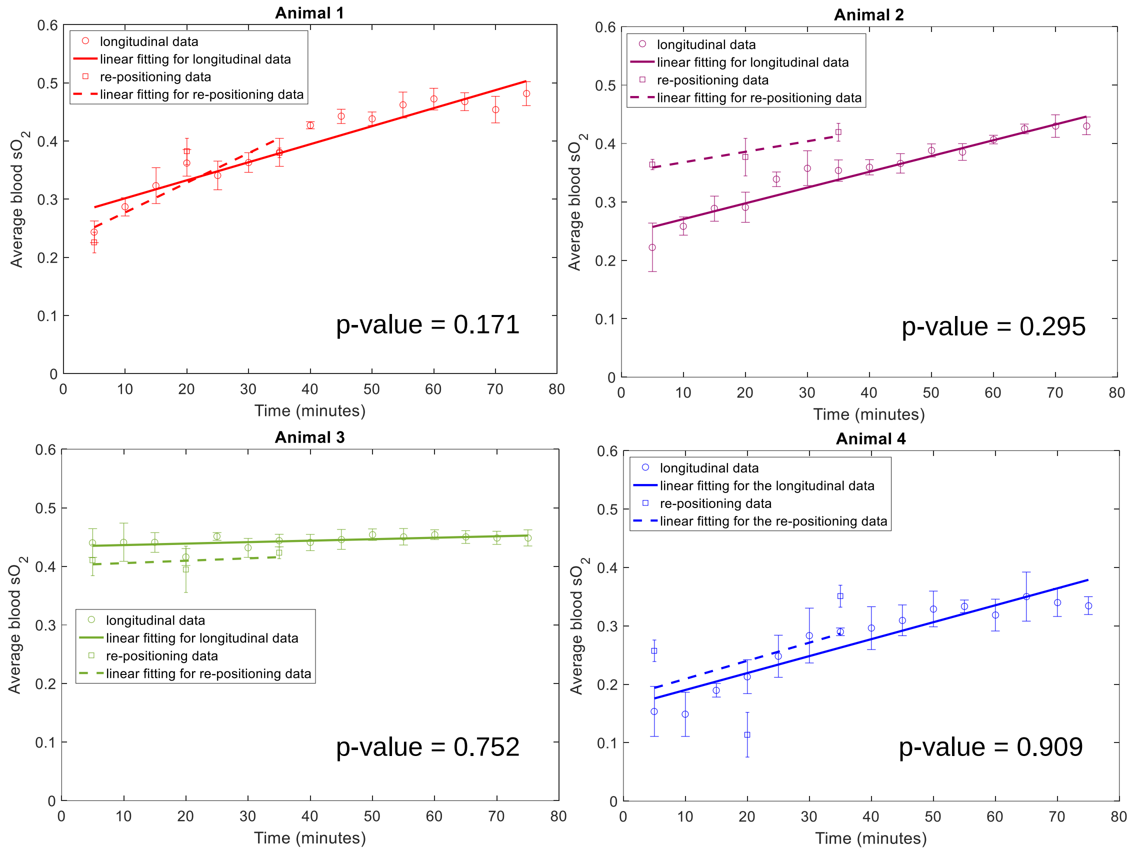

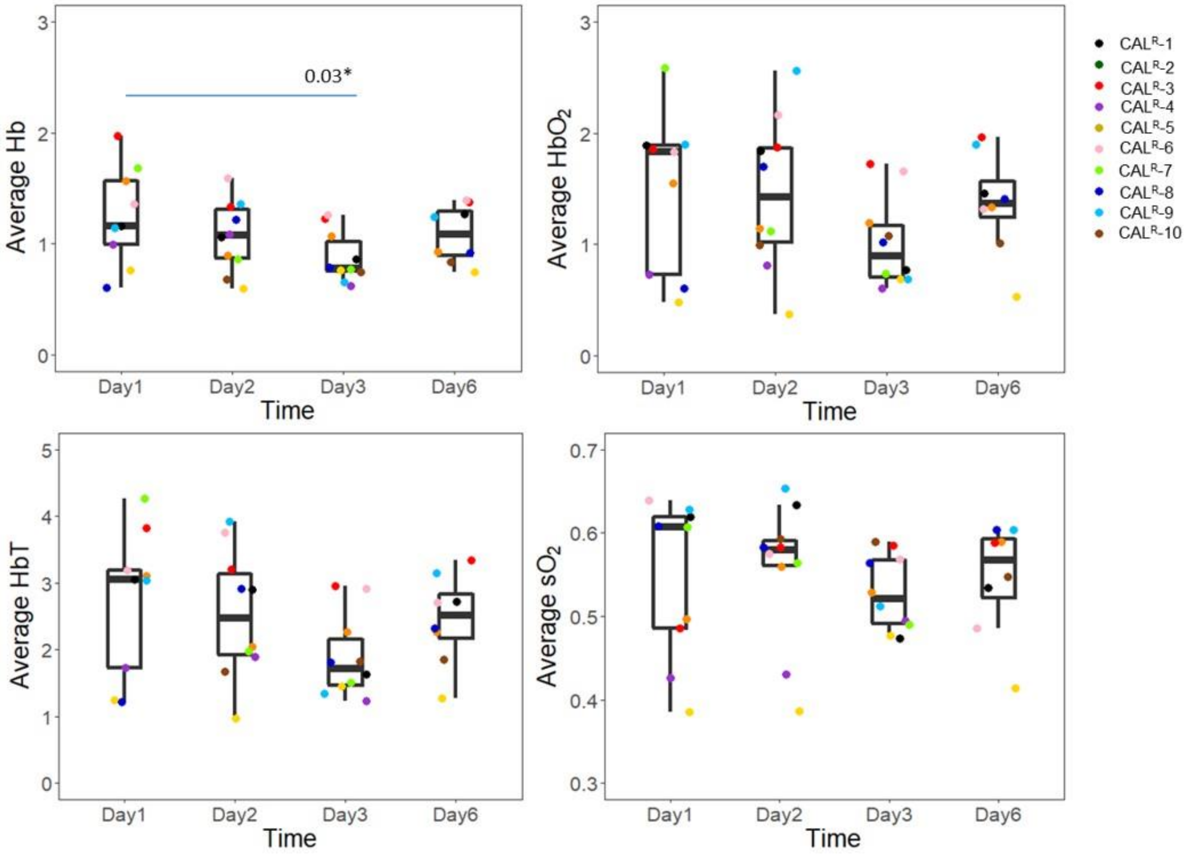


**Fig. S7:** Average haemoglobin and blood $sO_2$ values for the 3 central tumour slices of 10 CAL$^R$ tumours, over a 6 day imaging period, for air-breathing imaging. Day 1 corresponds to the time at which tumours reached approximately 200 mm$^3$. The whisker plots shows the distribution of the measurements; the central black line in each box represents the median of the population; top and bottom horizontal lines in each box represent the upper and lower quartile and the whiskers the minimum and maximum values.

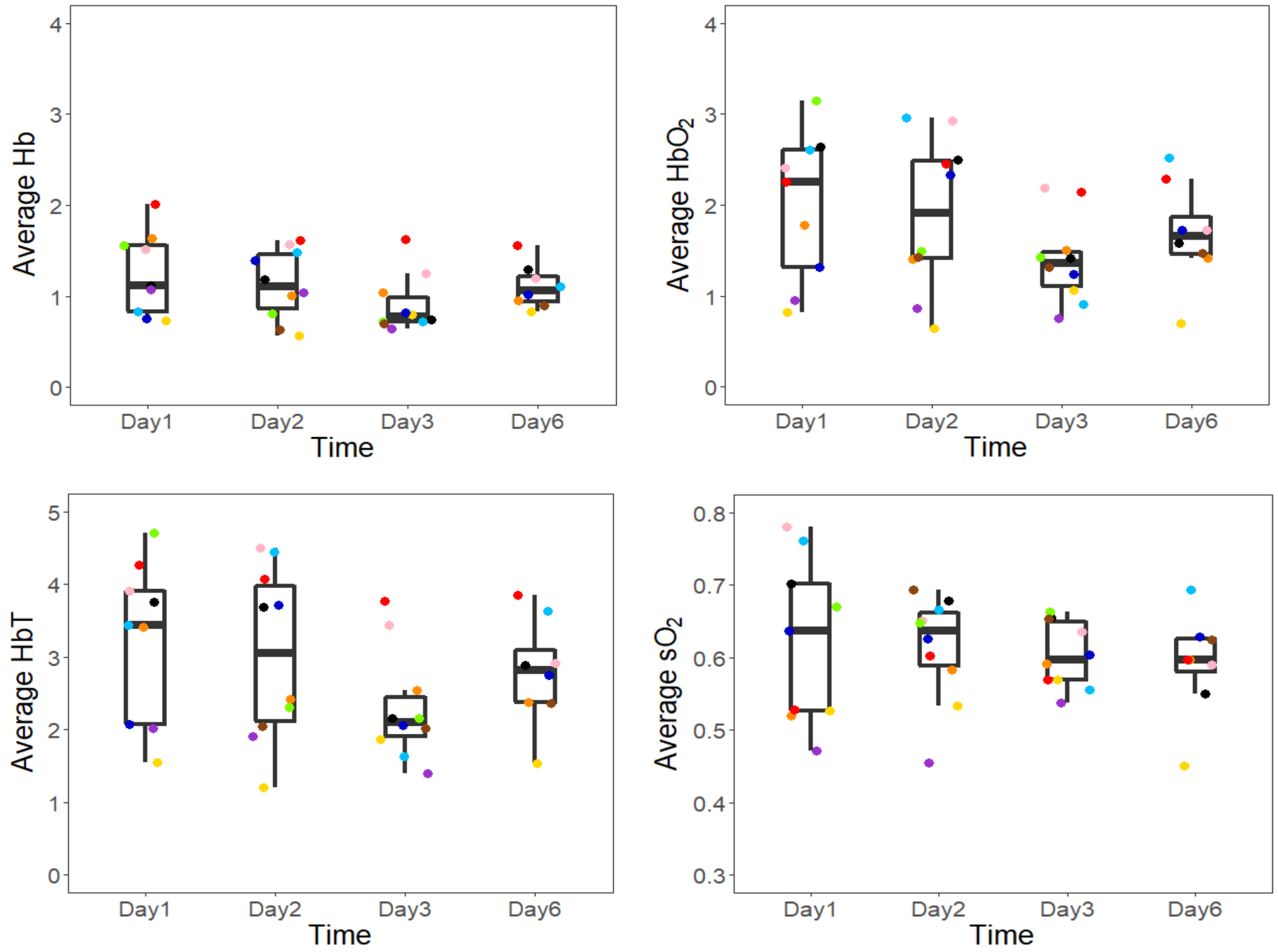


**Fig. S8:** Average haemoglobin and blood $sO_2$ values for the 3 central tumour slices of 10 $CAL^R$ tumours, over a 6 day imaging period, for oxygen-breathing imaging. Day 1 corresponds to the time at which tumours reached approximately 200 $mm^3$. The whisker plots shows the distribution of the measurements; the central black line in each box represents the median of the population; top and bottom horizontal lines in each box represent the upper and lower quartile and the whiskers the minimum and maximum values.

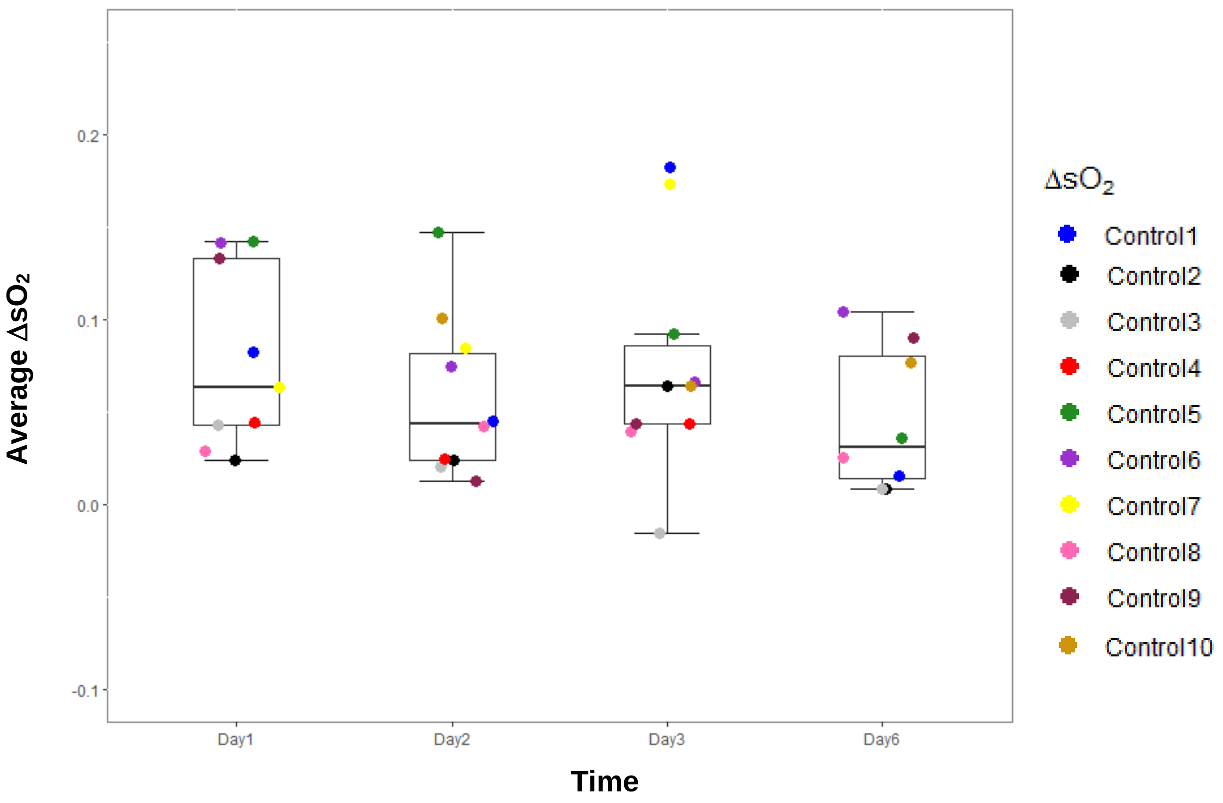


**Fig. S9:** Average blood $\Delta sO_2$ values for the 3 central tumour slices of 10 CAL[R] tumours, over a 6 day imaging period. Day 1 corresponds to the time at which tumours reached approximately 200 $mm^3$. The whisker plots shows the distribution of the measurements; the central black line in each box represents the median of the population; top and bottom horizontal lines in each box represent the upper and lower quartile and the whiskers the minimum and maximum values.

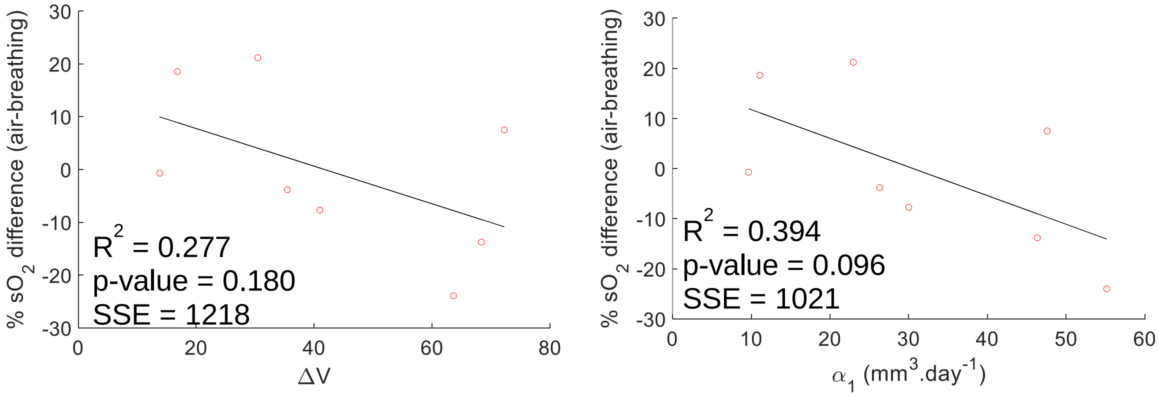


**Fig. S10:** Correlation between rate of change in volume between day 1 and 6 of imaging, $\Delta V$, or linear growth rate, $\alpha 1$, and the percentage difference in $sO_2$ between days 1 and 6 of imaging for 8 CAL[R] tumours, for air-breathing measurements.
